# Hydrogen reduction of molybdenum oxide at room temperature

**DOI:** 10.1038/srep40761

**Published:** 2017-01-17

**Authors:** Andreas Borgschulte, Olga Sambalova, Renaud Delmelle, Sandra Jenatsch, Roland Hany, Frank Nüesch

**Affiliations:** 1 Empa, Swiss Federal Laboratories for Materials Science and Technology, Laboratory for Advanced Analytical Technologies, Überlandstrasse 129, CH-8600 Dübendorf, Switzerland; 2 University of Zürich, Department of Chemistry, Winterthurer Strasse, 190, CH-8057 Zürich, Switzerland; 3 Institute of Materials and Process Engineering (IMPE), ZHAW - Zürcher Hochschule für Angewandte Wissenschaften, Technikumstrasse 9, CH-8400 Winterthur, Switzerland; 4 Empa, Swiss Federal Laboratories for Materials Science and Technology, Laboratory for Functional Polymers, Überlandstrasse 129, CH-8600 Dübendorf, Switzerland; 5 Institut des Matériaux, Ecole Polytechnique Fédéral de Lausanne, EPFL Station 12, CH-1015 Lausanne, Switzerland

## Abstract

The color changes in chemo- and photochromic MoO_3_ used in sensors and in organic photovoltaic (OPV) cells can be traced back to intercalated hydrogen atoms stemming either from gaseous hydrogen dissociated at catalytic surfaces or from photocatalytically split water. In applications, the reversibility of the process is of utmost importance, and deterioration of the layer functionality due to side reactions is a critical challenge. Using the membrane approach for high-pressure XPS, we are able to follow the hydrogen reduction of MoO_3_ thin films using atomic hydrogen in a water free environment. Hydrogen intercalates into MoO_3_ forming H_x_MoO_3_, which slowly decomposes into MoO_2_ +1/2 H_2_O as evidenced by the fast reduction of Mo^6+^ into Mo^5+^ states and slow but simultaneous formation of Mo^4+^ states. We measure the decrease in oxygen/metal ratio in the thin film explaining the limited reversibility of hydrogen sensors based on transition metal oxides. The results also enlighten the recent debate on the mechanism of the high temperature hydrogen reduction of bulk molybdenum oxide. The specific mechanism is a result of the balance between the reduction by hydrogen and water formation, desorption of water as well as nucleation and growth of new phases.

With oxygen having a much larger electronegativity than hydrogen, most binary d-metal oxides are much more stable than the corresponding hydrides^1^,^2^. Exposed to a gas mixture even at low hydrogen to oxygen ratios of 1:10^−6^, most transition metals form binary oxides instead of ternary hydrido-oxides or oxy-hydrides. Hydroxides do exist, but tend to decompose into the corresponding oxides as well^3^. However, due to the small atomic diameter and the ambivalent character of hydrogen, hydrogen can intercalate into oxides and act as a donor or acceptor of electrons^4^,^5^,^6^, being the origin of various optical and electronic effects with many applications.

The effect is particularily strong in WO_3_^7^,^8^ and MoO_3_^9^,^10^, which have long been known for their gasochromic properties, and can be utilized as hydrogen^10^ and ammonia sensors^11^. Hydrogen atoms from molecular hydrogen or decomposition of hydrogen containing reducing molecules such as ammonia intercalate into the oxide and form hydrogen molybdenum bronzes H_x_Mo_x_^5+^Mo_1−x_^6+^O_3_^10^. The phase transformation causes only small crystallographic rearrangements (topotactic reduction): hydrogen atoms occupy sites in the van der Waals gaps between double layers of MoO_6_ octahedra as well as intralayer sites on zigzag chains along the channels^12^,^13^. This results in a relatively small increase of the cell volume and slight distortion of the lattice changing the overall crystal symmetry from orthorhombic orthorhombic (phase I) to monoclinic (phases II, IV)^12^,^14^. Although lattice distortion and hydrogen ordering increase the complexity of the system^12^, the electronic structure may be described using the semiconductor model with hydrogen as dopant (Fig. 1). In this model, the MoO_3_ structure motif (MoO_6_ octahedrons) remains unchanged and defines the overall electronic structure, but is perturbed by hydrogen atoms as new band gap states^10^. These states change the optical and electrical properties: pristine MoO_3_ consists of Mo^6+^ forming the conduction bands and O^2−^ forming the valence bands. Pristine MoO_3_ is semiconducting (and thus transparent) with a band gap of 3.2 eV^10^. Hydrogen gives its electron to the conduction band forming protons and “valence band-like Mo^5+^ states” (see Fig. 1: green/grey colored states in the band gap). As a result, a hydrogenated MoO_3_ film appears blue due to the intervalence-charge transfer from the newly formed Mo^5+^ to adjacent Mo^6+^ upon optical excitation. The controversy remains, whether the optical properties are sufficiently described by the intervalence charge transfer theory between Mo^5+^ and Mo^6+^ ions, or by polaron absorption (small-polaron theory)^10^, which nonetheless relies on the formation of Mo^5+^ states. Simultaneously with the color change, the electrical conductivity increases, supporting the doped semiconductor model. There is a maximum doping level of hydrogen: exceeding *x* = *1* in H_x_MoO_3_, leads to formation of Mo^4+^ ions (sketched by the width of the electronic states in Fig. 1). However, this situation seems to be unstable. Indeed, at higher concentrations water and hydrogen free MoO_2_ consisting of Mo^4+^ and O^2−^ states is formed. Every missing oxygen atom is balanced by two more electrons in the Mo 4d bands (see Fig. 1). This picture explains the effect of oxygen defects on the electronic structure of the MoO_3_ phase as well. The phase transformation is associated with major structural rearrangements and correspondingly high activation barriers. Thus, it is usually only observed at high temperatures or at very high driving forces [see, e.g., ref. 15, and discussion later]. The precursor of this transformation may be the formation of OH ions as observed for hydrogen concentrations *x* ≫ *1* by inelastic neutron scattering^13^. The ambivalent character of hydrogen is similar in the related WO_3_ system: its electronic structure analogous to the one of MoO_3_ sketched in Fig. 1 depends sensitively on the fact whether H-O-H bridge bonds are formed, or whether hydrogen acts as a delocalized dopant in the semiconductor^16^.

In addition to the changes observed during exposure to hydrogen gas, MoO_3_ is photo- and electrochromic, and thus used for photo- and electrochromic coatings^10^. Gasochromism and photo/electrochromism are directly related: optically or electronically excited electrons in the conduction band can decompose water adsorbed at surface and interfaces of MoO_3_. The produced hydrogen intercalates into the bulk and changes the optical and electronic properties of MoO_3_ as describe above^10^.

Thin MoO_3_ films can be quite simply prepared by physical^11^ and chemical vapor deposition^17^ or via coating-processes using precursor solutions^18^ or nanoparticle dispersions^19^. Thus, MoO_3_ has been proposed as an anode interfacial layer in organic^16^,^20^,^21^,^22^,^23^,^24^ and dye-sensitized solar cells^24^. The low charge injection/extraction barrier at MoO_3_/organic interfaces is believed to be due to the favorable energy level alignment between the high work function value of MoO_3_ and the highest occupied molecular orbital of an organic molecule^21^,^22^,^23^. Due to the complex chemistry of this material and the strong impact of external stimuli on the electronic properties, a coherent understanding of the hole extraction process across MoO_3_ in OPV cells is still lacking^24^. For the application as an electron injection/extraction layer, the formation of hydrogen bronzes by photolysis of hydrogen containing molecules is an undesired effect, because it diminishes the favorable electronic properties^24^.

Hydrogen bronzes of MoO_3_ are metastable states^26^: they can be further reduced via formation of water to lower oxides and thus the oxygen – metal ratio depends on the specific experimental conditions. In gasochromic and related applications, the hydrogen concentration is relatively high, and oxygen (water) can leave the oxide resulting in irreversible reduction of the latter to MoO_2_, and eventually to Mo metal. The easy reducibility can thus be hypothesized to be the origin of the limited reversibility of such devices. On the other hand, it is assumed to be the source of the remarkable catalytic properties for oxidation reactions of hydrocarbons and alcohols^27^.

Because of its evident importance, bulk as well as surface properties of MoO_3_ have been carefully studied by surface science methods such as photoemission and electron diffraction and bulk methods (*e.g.,* XRD, EXAFS)^15^,^28^. Simplified, the outcome is as follows: at higher temperatures (>700 K), the surface of MoO_3_ is reduced already in vacuum^29^. Reduction of bulk MoO_3_ requires high temperature and hydrogen at several mbars, and is proportional to the hydrogen partial pressure^28^. The exact decomposition pathway from MoO_3_ to eventually Mo metal, in particular the significance of intermediate phases such as Mo_4_O_7_, is still controversially debated. This is due to unprecise experimental conditions (hydrogen purity, in particular the water/oxygen contaminations), and the number of possible reaction steps involved: hydrogen dissociation, hydrogen diffusion, water formation and diffusion, phase nucleation and growth^28^. Furthermore, the high temperature results are not directly transferable to room temperature, as the rate limiting step may change.

In this paper, we apply the XPS-membrane approach^30^ to analyze the reduction mechanism of MoO_3_ thin films at room temperature. X-ray photoelectron spectroscopy (XPS) is an ideal tool to measure the oxidation states of the elements in oxides, see, e.g. ref. 31, and has been successfully used to study MoO_3_^23^,^32^,^33^. Unlike in UHV-XPS-systems, where only *post-mortem* analysis of *ex-situ* treated films is possible, this approach allows an operando study of the hydrogenation of MoO_3_ at relevant hydrogen pressures^32^. For this, the MoO_3_ thin film is deposited on a hydrogen permeable Pd-membrane (see Fig. 2). The high selectivity for hydrogen diffusion in Pd guarantees an ultrapure quality of hydrogen entering the MoO_3_. Furthermore, it is atomic hydrogen interacting with the oxide. The dissociation of hydrogen on oxide surfaces depends on various physico-chemical parameters (among them the oxidation state of the metal). Therefore, the hydrogen flux into the sample of a bare oxide thin film is difficult to control, in contrast to the membrane approach, where it is controlled by adjusting the hydrogen pressure at the back of the membrane. Hydrogen dissociation on Pd is fast as is the subsequent hydrogen diffusion through the Pd-membrane. Possible rate limiting steps of the reduction of MoO_3_ are now limited to oxygen diffusion (desorption) and nucleation and growth of new phases. Furthermore, the approach mimics the situation in gasochromic sensors, because also here a Pd layer on top of the thin film provides atomic hydrogen interacting with the layer. The same is true for electrochromic and photochromic devices: water splitting by electrons or photons results in atomic hydrogens first. Surface science methods are usually limited to UHV-pressures, the membrane approach allows pressures up to 1 bar hydrogen^30^. In addition, the membrane provides atomic hydrogen, i.e., hydrogenation of catalytically inactive surfaces such as oxides is possible as well.

## Results

Figure 2 shows typical XPS-overview spectra of MoO_3_ deposited on a Pd membrane as prepared and after long term hydrogenation. The film thickness of 10 nm is just thick enough to consider it behaving bulk-like (the interfaces of noble metals to MoO_3_ are discussed in depth in ref. 21). From the peak intensities of the oxygen 1 s and molybdenum 5d peaks, the metal/oxygen stoichiometry is determined^33^. In good agreement with literature, the oxygen content decreases after hydrogenation. However, the oxygen loss is not directly related to the applied hydrogen pressure. This is indicated by the time dependence of the process. The diffusion through the Pd-membrane is relatively fast: in previous measurements on hydrogen permeation through the 150 μm thick Pd membrane we observed dynamic equilibrium after approximately 500 s (see Methods section, ref. 30) due to the fast hydrogen diffusion in Pd even at room temperature. We do not expect deceleration by hydrogen diffusion through the 10 nm thin MoO_3_ film (the diffusion parameter of H in MoO_3_ is of the order of 10^−11^ m^2^/s^29^,^34^). However, after an initial hydrogenation at 100 mbar, vaccum was applied. Unexpectedly, a decrease of the oxygen content was measured after hours in vacuum (Fig. 3). The rate limiting step is thus not hydrogen diffusion.

More details are visible after magnifying the spectral regions of the Mo 5d and O 1 s core levels. Figure 3 compares the oxygen 1 s core levels as deposited and after long term exposure to hydrogen. The data is fitted to two lines separated by approximately 1.6 eV. The value of the main peak most prominently visble in the as deposited MoO_3_ film is in perfect agreement with literature, see, e.g. refs 33,35. The second peak may be attributed to oxygen as OH in MoO_3_ following the discussion of the electronic structure. However, prior to desorption, water formed by the hydration of MoO_3_ will accumulate at the surface. The existence of the peak in the as deposited state as well as the observed oxygen loss, which proceeds via the desorption of water, supports the interpretation of this peak as water adsorbed at the surface^20^. A better probe for the evolution of the electronic structure during hydrogenation are the Mo 5d states. Figure 4 shows the evolution of the Mo 5d states during hydrogenation at 100 mbar. The shape and positions of the peaks change drastically indicating the change of the oxidation state of Mo from Mo^6+^ to Mo^4^ ^20^,^23^,^32^,^33^,^35^.

A fitting of such complex line shapes is error prone if performed on single spectra alone. However, the power of the membrane approach is the possibility to follow the evolution of the electronic structure *operando*. Starting situation is defined: Mo^6+^ states exhibiting two 5d core excitations (5/2 and 3/2). Low hydrogen dose results in additional peaks assigned to Mo^5+^. With continued hydrogenation, a shoulder at lowest binding energy develops, which is assigned to Mo^4+^. To corroborate the formation of Mo^4+^-states, we compare the reduced MoO_3_ spectrum with literature data (see Methods Section). All oxidation states display two peaks^20^,^22^,^32^,^33^,^35^. To resolve the spectra, we fit all 6 peaks to predefined fixed binding energies and widths; and the intensities, *i.e.*, only the concentrations are chosen as free fitting parameters. This causes a slight scattering of the fitting results, because small deviations of the exact binding energies as a function of the hydrogen concentration are not included. However, the outcome is very clear: the growth of the Mo^5+^ states takes place at the expense of the Mo^6+^ states. The corresponding kinetics is much slower than the diffusion of hydrogen (see discussion above, Methods section). The rate limiting step is thus not the hydrogen supply, nor desorption of water (oxygen, see Fig. 2), and is therefore assigned to the nucleation and growth of the new H_x_MoO_3−δ_ phase.

Surprisingly, the Mo^4+^ state develops independently from this behaviour, and linearly increases over time (Fig. 4). This was also confirmed by experiments in which the hydrogen backpressure at the membrane was set to zero, but the evolution of the Mo^4+^ states and loss of oxygen (see Fig. 2), i.e., the formation of a molybdenum oxide MoO_x_ with an oxygen content of *x* ≪ 3, continued. As discussed in the Methods section, the high chemical potential of hydrogen in the MoO_3_ films is reached on a much faster time scale than the observed evolution of the Mo^4+^-states. Thus, the supply of hydrogen is not the rate-limiting step. The oxygen content of the new phase associated with Mo^4+^ is lower than in the hydrogen bronze, and thus oxygen removal is rate-limiting for forming this phase. Due to the noise in the data, only the trend of decreasing oxygen content is experimentally verified. Locally higher oxygen content at the surface may also be realized by reshuffling of oxygen by phase separation into oxygen rich and pure phase as suggested in ref. 33. It is worth noting that the reduced state once reached does not reverse back to the original state in vacuum, at least within the measurement time of several days.

## Discussion

These results shed light on the kinetic behaviour of the phase formation in non-equilibrium conditions. Numerous studies investigate the occurrence of intermediate phases during (high-temperature) reduction to unravel the decomposition mechanism. In particular the one-step reduction mechanism (MoO_3_ directly to MoO_2_) is discussed against multiple step mechanisms with additional intermediates being involved (*e.g*. Mo_4_O_11_^15^). To simplify the discussion, we have sketched a qualitative potential energy surface (PES) for the reduction reaction of MoO_3_ to MoO_2_ in Fig. 5. The hydrogen bronze is a thermodynamically unstable state^26^, which will eventually be further reduced because of the globally lower free energy of MoO_2_ and water^1^,^15^,^26^. However, this 2^nd^ reduction step is associated with the removal of oxygen by water. For both steps, an energy barrier has to be overcome. As the systems has a high number *n* of degree of freedom (reaction coordinates), the PES is a *n*-dimensional hyper surface, simplified as a 2-dimensional surface in Fig. 5. While energy minima can be related to thermodynamic parameters, the barriers and thus the reaction path requires complex calculations of the electronic structure. The potential energy surface displayed here is an educated guess based on empirical figures. We give the enthalpy of formation of the various phases expected to occur to illustrate their location on the energy potential surface shown in Fig. 5 (data from ref. 26). As a quantitative indicator for the barrier heights, we added the activation energies of reduction at higher temperatures (data from ref. 15). It is worth noting that also the experimentally derived activation energies depend on the pathway.

**Figure 1:**
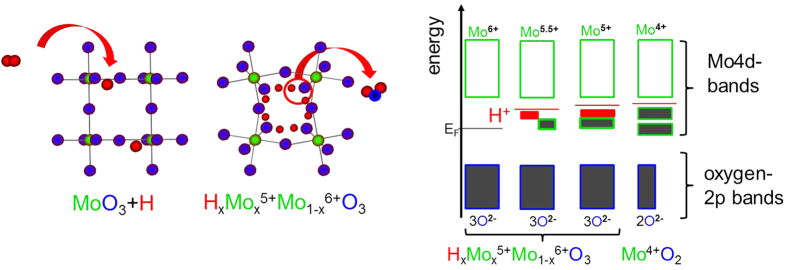
Left: Simplified crystal structure of MoO_3_ and hydrogen bronze(s). Chains of MoO_6_ octahedrons are fused together by edge sharing to form corrugated layers. The intercalation of hydrogen into MoO_3_ does not markedly modify the structural motif apart from slight increase of the cell volume, lattice distortion and hydrogen ordering^12^,^13^,^14^. As already suggested by the vicinity of hydrogens to oxygen, water is formed at higher concentrations. In this case, the metal/oxygen ratio is modified leading to MoO_2_. The changes of the electronic structure by hydrogen intercalation are depicted in the right panel. Hydrogen forms band gap states (red) and “valence band-like Mo^5+^ states” (green-rimmed boxes: grey: occupied, white: empty), as depicted by the width of electronic states. The loss of oxygen at higher hydrogen concentrations is depicted by the smaller width of the blue rimmed boxes, simultaneously, more Mo bands are filled.

**Figure 2:**
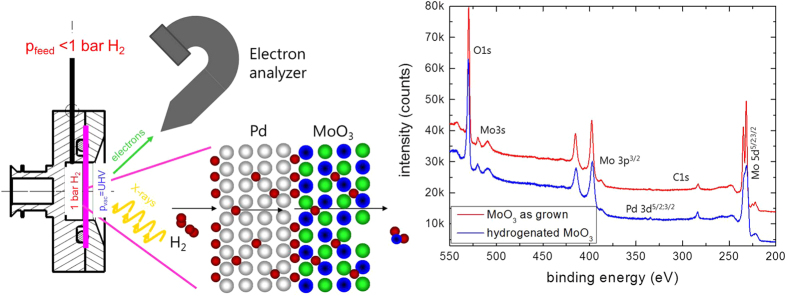
Left panel: membrane approach for XPS measurements. Right panel: XPS-overview spectra of as deposited MoO_3_ before and after hydrogenation. See also Methods section.

**Figure 3:**
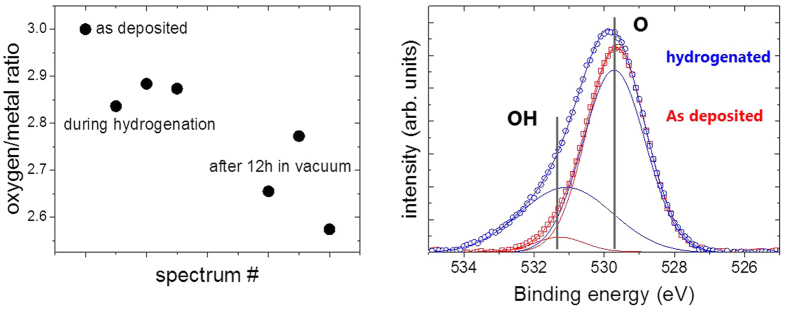
Left panel gives the oxygen/metal ratio during and after hydrogenation at 100 mbar. The right panel shows details of the oxygen 1 s core level spectra as deposited and after long term exposure to hydrogen (background subtracted). The data is fitted to two lines centered around 529.6 ± 0.2 eV and 531. 2 ± 0.2 eV, assigned as O and OH, respectively. The intensity ratio between OH and O peaks increases from approximately 6% to 50%.

**Figure 4:**
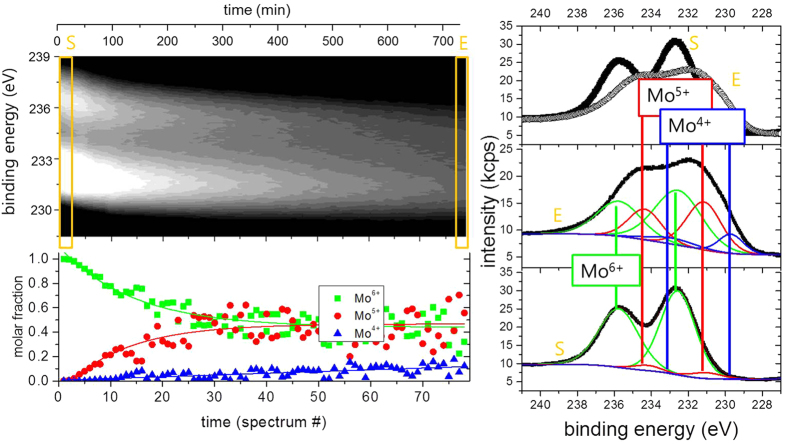
Time resolved XPS-measurements of the Mo 5d states during hydrogen intercalation (hydrogen pressure 100 mbar). The grey-scale image (left top image) is a 2D representation of the time evolution. In the right graph, the starting (S) and final (E) spectra are plotted and the applied fits considering Mo^6+^, Mo^5+^, and Mo^4+^ 5d 5/2 and 3/2 states are given in the bottom left image. Their intensities are taken as measure for the molar fraction of the respective phases shown with the grey-scale plot.

The results at hand also indicate that the reaction pathway depends on the rate limiting step controlled by the external conditions: in our experiments, the chemical potential of hydrogen supply is very high, and thus the system follows the kinetically favoured path: that is, the intercalation of hydrogen due to the small activation energy 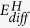 for hydrogen diffusion in MoO_3_ (see Fig. 5, ref. 29) and thus formation of hydrogen bronzes. However, the lower reduction state Mo^4+^ is simultaneously formed, but at a much slower pace (compare *E*_*A*_ and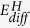 in Fig. 5). Oxygen deficient MoO_3_ surfaces, which can be imagined as MoO_2_ inclusions in a MoO_3_ matrix, can be formed by heating in UHV without further reductant^33^. As this takes place only at high temperatures (large *E*_*A*_), co-existing phases will be observed only during low temperature reduction. Furthermore, the multiple steps may not be consecutive steps. The hydrogen bronze is a meta-stable phase, i.e., hydrogen absorption without water formation is exothermic^26^, but further reduction under formation of water is thermodynamically favoured (Fig. 5)^1^. In addition to the transport of atoms, the kinetics of crystalline phase transformations are limited by long-range order effects. Although the change of the character of hydrogen in the oxide from a delocalized proton to a localized atom bridged to oxygen atoms proceeds without major structural reorientations (compare Fig. 1), the corresponding changes of the overall electronic structure affect the whole crystal.

The local energy minimum of H_x_MoO_3_ explains its practical existence, but bronzes with high hydrogen concentrations are only formed under high chemical hydrogen potentials using atomic hydrogen from chemical reactions (nascent hydrogen), gaseous hydrogen dissociated at Pd overlayers (typical hydrogen sensor setup), or produced electrochemically^9^,^12^,^26^, where water removal is slow. The formation of further reduced oxide and water is thermodynamically preferred, explaining the observed irreversibility of the membrane-hydrogenated MoO_3_ thin films. Catalysts usually consist of MoO_3_ particles, which are practically inert at low temperature (Fig. 5). The formation of oxygen deficient surfaces at high temperature enables hydrogen dissociation, and also desorption of water is faster at high temperature. In this case, direct reduction of MoO_3_ to MoO_2_ without formation of a bronze is possible. Similar qualitative explanations are valid for the existence of the various other crystalline phases observed during reduction.

The analysis gives guidelines for better reversibility of devices made of MoO_3_. The reduction of Mo should take place without removal of oxygen. This implies the encapsulation of the MoO_3_ by materials, which do not getter oxygen. In hydrogen sensors, this is less a question of the choice of materials (typical setup: substrate/MoO_3_/Pd^10^, Fig. 5), but of the quality of the thin film structure (roughness, coverage)^11^. The conditions in organic solar cells are particularly difficult: here, MoO_3_ interfaces organic semiconducting small molecules or polymers, possibly even containing residual organic solvents^36^. Most of the chemicals used are reducing and hygroscopic, and thus add an additional thermodynamic driving force for the reduction of MoO_3_ via water formation, which affects the device performance^36^. Finally, we note that the experimental observation of low temperature reduction to Mo^4+^ is possible only using the membrane approach guaranteeing high flux of hydrogen atoms into the sample and providing a free surface for water desorption.

To summarize, we demonstrated the *operando* XPS analysis of the reduction of MoO_3_ by hydrogen, from which we derived the reaction pathway of the reduction around room temperature. The low temperature results join the missing link between reaction mechanisms proposed for bulk materials at high temperature hydrogenation and the hydrogen-oxide interactions. We show that several reaction pathways of the hydrogen reduction take place simultaneously. The preference of one of these pathways can be controlled by the experimental conditions (open/closed setup, exposure to atomic/molecular hydrogen, temperature), but the then subdominant pathway may still occur as side reaction with detrimental effects on the performance of the sought application. *E.g*., in hydrogen sensors, the reversible intercalation in and out of MoO_3_ is competing with the unwanted irreversible water formation, which eventually disables the sensor function. This situation is observed in many related materials systems such as WO_3_ with a broad range of applications from optically active thin films^10^ and chemical sensors^10^,^11^, hole extraction layers in PV^17^,^37^, catalysis^27^, to solar water splitting^38^. An important factor in these applications, which was addressed here for the MoO_3_ system, is the transient nature of the various states of matter during hydrogen exposure. The time constant in hydrogen W/Mo bronzes is relatively long; however, there are indications of short-lived, but highly mobile and reactive hydrogen states in various other meta-stable systems^4^, to be studied using the membrane approach.

## Methods

10 nm thick MoO_3_ thin films were deposited onto 150 μm thick polycrystalline Pd-membranes by thermal evaporation (0.2 Å/s). *In situ* XPS surface analysis was performed in a modified VG EscaLab spectrometer with a base pressure below 10^−9^ mbar. XPS spectra were collected with a SPECS PHOIBOS 100 analyzer using a non-monochromatic X-ray source (Al K alpha: 1486.6 eV).

**Figure 6:**
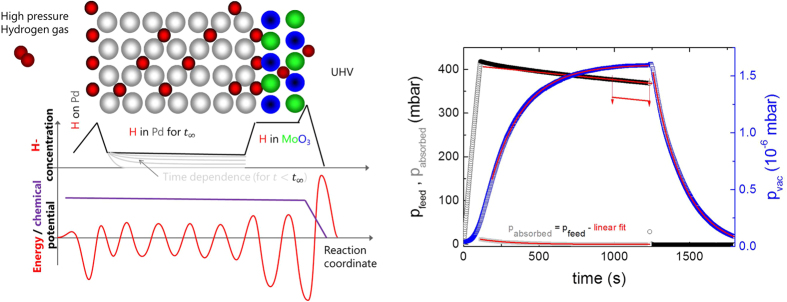
The chemical potential of hydrogen in MoO_3_ on Pd-membranes can be considered to be nearly constant, although the hydrogen concentration varies going from Pd high pressure feed through the membrane to the top of the MoO_3_ surface. The concentration in a material depends on the hydrogen solubility in the bulk of the membrane setup, and on the chemisorption enthalpy on its surfaces (as sketched by a simplified energy potential diagram). However, the chemical potential is balanced by the corresponding entropy terms, so that it is continuous. This is perfectly true for equilibrium conditions. The membrane works in quasi-equilibrium. However, because of the low desorption probability of H_2_ from MoO_3_ due to the high activation barrier, while absorption of hydrogen on the feed side is facilitated by the small activation energy of hydrogen dissociation on Pd, this quasi-equilibrium is near to equilibrium. In kinetic experiments as performed here, quasi-equilibrium is reached within a finite time, which depends on the amount of hydrogen absorbed in the bulk of the membrane (depicted by grey lines). The right graph shows the time dependence of the pressure at the feed side, which can be converted into the amount of hydrogen entering the membrane, and the pressure on the UHV side. A linear decrease of the feed pressure (corresponding to a constant UHV-pressure) is indicative of a constant flux through the membrane, which is reached within 500 s. See ref. 30 for more details.

**Figure 7:**
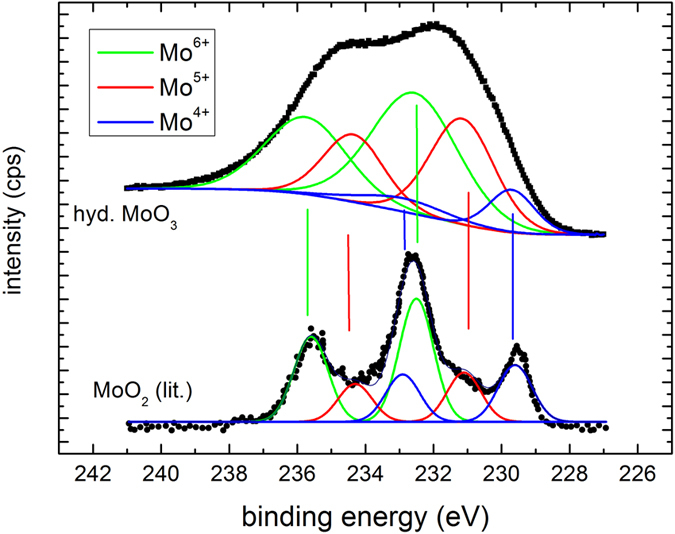
XPS-measurement of the Mo 5d states after long term (hydrogen pressure 100 mbar) and of MoO_2_ (literature data, ref. 39). The quality of the fits to the literature data is worse because of digitalization.

The membrane approach is based on a relatively simple setup to overcome the so-called pressure gap in photoemission. The core idea of the approach is the membrane device, which is exposed to UHV in the analysis chamber on one side and ambient pressure hydrogen on the other. The hydrogen flux from the surface into the vacuum is desorption rather than diffusion limited, leading to the equivalence of ambient pressure conditions at the analysed surface of the membrane (Fig. 6). For details of the membrane approach we refer to ref. 30. Data evaluation was performed using CasaXPS. For fitting of the Mo 5d states, we used the following parameters:


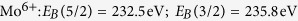



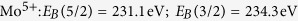



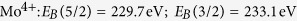


in good agreement with literature data^32^,^33^,^35^,^39^. The intensity ratio of the 5/2 to 3/2 peak was fixed to 5/3, which is also very near to the result obtained from fitting using free parameters of as prepared MoO_3_ spectra consisting of only 6+ states. Figure 7 is a comparison of measurements on membrane-hydrogenated MoO_3_ and literature data on MoO_2_ (from ref. 39). Although MoO_2_ may be described by one Mo^4+^ and two O^2−^ ions, the XPS shows all three states^38^.

## Additional Information

**How to cite this article**: Borgschulte, A. *et al*. Hydrogen reduction of molybdenum oxide at room temperature. *Sci. Rep.* **7**, 40761; doi: 10.1038/srep40761 (2017).

**Publisher's note:** Springer Nature remains neutral with regard to jurisdictional claims in published maps and institutional affiliations.

## Figures and Tables

**Figure 5:**
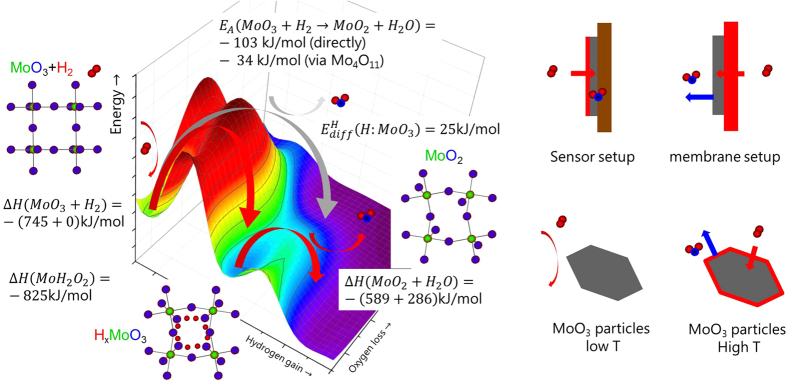
Left: Sketch of the potential energy surface for the reduction of MoO_3_ to MoO_2_ to depict the possible reaction pathways. The z-axis is the energy coordinate, while x- and y-axes represent the not further defined reaction coordinates. MoO_2_ is most stable with respect to MoO_3_ and hydrogen, however, hydrogen bronze H_x_MoO_3_ is a metastable state. The corresponding enthalpies of formations serve as quantitative estimates for the position on the energy potential surface^26^. The activation energy of the reactions depends on the path over the potential energy surface. The corresponding experimental activation energies *E*_*A*_ for the high temperature reduction are indicated^15^. Hydrogen diffusion is relatively fast in MoO_3_, which results from the small activation energy for diffusion 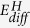
29. The latter is related to the electronic structure on one hand, but the reaction path is also affected by the external conditions temperature, hydrogen and water partial pressure on the other. These conditions depend on the experimental setup as sketched in the right figures: catalysts are usually made of MoO_3_ particles, which are basically inert at room temperature, i.e., they do not dissociate hydrogen nor is desorption of water facilitated (bottom left). At higher temperature, an oxygen deficient, dissociatively active surface is formed with high oxygen mobility (bottom right). To overcome the high dissociation at room temperature, one can coat a MoO_3_ thin film on an inert substrate with Pd (typical sensor setup, top left). In our membrane approach, the MoO_3_ thin film is hydrogenated via the Pd substrate, i.e., potentially formed water can leave the film.
